# Changes in shunt, ventilation/perfusion mismatch, and lung aeration with PEEP in patients with ARDS: a prospective single-arm interventional study

**DOI:** 10.1186/s13054-020-2834-6

**Published:** 2020-03-23

**Authors:** Dan Stieper Karbing, Mauro Panigada, Nicola Bottino, Elena Spinelli, Alessandro Protti, Stephen Edward Rees, Luciano Gattinoni

**Affiliations:** 1 Respiratory and Critical Care Group, Department of Health Science and Technology, Aalborg University, Fredrik Bajer Vej 7E, DK-9220 Aalborg East, Denmark; 2 Dipartimento di Anestesia, Rianimazione (Intensiva e Subintensiva) e Terapia del Dolore, Fondazione IRCCS Ca’ Granda - Ospedale Maggiore Policlinico, Milan, Italy; 3 Department of Anesthesiology, Emergency and Intensive Care Medicine, University of Gӧttingen, Gӧttingen, Germany

**Keywords:** ARDS, PEEP, CT, Lung aeration, Shunt, Ventilation/perfusion mismatch

## Abstract

**Background:**

Several studies have found only a weak to moderate correlation between oxygenation and lung aeration in response to changes in PEEP. This study aimed to investigate the association between changes in shunt, low and high ventilation/perfusion (V/Q) mismatch, and computed tomography-measured lung aeration following an increase in PEEP in patients with ARDS.

**Methods:**

In this preliminary study, 12 ARDS patients were subjected to recruitment maneuvers followed by setting PEEP at 5 and then either 15 or 20 cmH_2_O. Lung aeration was measured by computed tomography. Values of pulmonary shunt and low and high V/Q mismatch were calculated by a model-based method from measurements of oxygenation, ventilation, and metabolism taken at different inspired oxygen levels and an arterial blood gas sample.

**Results:**

Increasing PEEP resulted in reduced values of pulmonary shunt and the percentage of non-aerated tissue, and an increased percentage of normally aerated tissue (*p* < 0.05). Changes in shunt and normally aerated tissue were significantly correlated (*r* = − 0.665, *p* = 0.018). Three distinct responses to increase in PEEP were observed in values of shunt and V/Q mismatch: a beneficial response in seven patients, where shunt decreased without increasing high V/Q; a detrimental response in four patients where both shunt and high V/Q increased; and a detrimental response in a patient with reduced shunt but increased high V/Q mismatch. Non-aerated tissue decreased with increased PEEP in all patients, and hyperinflated tissue increased only in patients with a detrimental response in shunt and V/Q mismatch.

**Conclusions:**

The results show that improved lung aeration following an increase in PEEP is not always consistent with reduced shunt and V/Q mismatch. Poorly matched redistribution of ventilation and perfusion, between dependent and non-dependent regions of the lung, may explain why patients showed detrimental changes in shunt and V/Q mismatch on increase in PEEP, despite improved aeration.

**Trial registration:**

ClinicalTrails.gov, NCT04067154. Retrospectively registered on August 26, 2019.

## Background

In acute respiratory distress syndrome (ARDS), computed tomography (CT) analysis has shown that recruitment followed by titrated positive end-expiratory pressure (PEEP) can improve lung aeration, supposedly by opening collapsed lung units and keeping them open [1].

PEEP is often adjusted according to oxygenation without CT measurement [2]. However, several studies have found only a weak to moderate correlation between oxygenation and aeration following PEEP changes [3–5]. The inconsistency between aeration and oxygenation may be due to the use of simple oxygenation parameters such as oxygen saturation or partial pressure related to inspiratory oxygen (PaO_2_/FiO_2_), as these parameters are sensitive to extrapulmonary factors such as ventilation and FiO_2_ [6, 7].

Studies using the reference technique for measurement of ventilation/perfusion (V/Q) mismatch including shunt and alveolar dead space, the multiple inert gas elimination technique (MIGET) [8], have demonstrated that hypoxemia in ARDS can be multifactorial, with some patients showing both large intrapulmonary shunt and significant perfusion of the lung regions with low V/Q ratios [9, 10]. MIGET studies have shown that, in general, increases in PEEP reduce the perfusion of shunted and low V/Q regions, but may worsen high V/Q mismatch [9–11]. These studies define much of the current understanding of the effects of PEEP on pulmonary gas exchange, but were not performed in combination with the investigation of lung aeration, and were carried out prior to the era of lung-protective ventilation. This means that high levels of tidal volume, pressures, and FiO_2_ were applied, all of which can have a significant impact on shunt and V/Q mismatch.

A simple bedside alternative to MIGET can estimate intrapulmonary shunt and degree of low and high V/Q mismatch from varying inspiratory oxygen and measuring end-tidal gasses, blood oxygenation, and the results of a single arterial blood gas sample [12]. This technique, also known as the ALPE method, is based on a model of pulmonary gas exchange and has successfully been applied in healthy subjects [13]; pre-, peri-, and postoperative patients [13–16]; and ICU patients including those with severe respiratory failure [7, 12, 13], and has been validated in comparison with the reference technique MIGET in animal models of homogeneous and inhomogeneous lung injury [17, 18]. The ALPE method considers both ventilation and perfusion, and as such, it may allow an improved explanation of the relationship between lung aeration, shunt, and V/Q mismatch, at different levels of PEEP.

To the best of the authors’ knowledge, no previous studies have compared CT scan measurement of lung aeration, with bedside, model-based estimation of shunt and ventilation/perfusion mismatch at different levels of PEEP during lung-protective ventilation in ARDS patients. The purpose of this preliminary study was therefore to explore whether PEEP-induced changes in pulmonary shunt and V/Q mismatch are correlated with changes in lung aeration in ARDS patients.

## Methods

### Study protocol

Thirteen patients were included in this prospective single-arm interventional study from August 2012 to August 2014 at the University Hospital of Fondazione IRCCS Ca’ Granda - Ospedale Maggiore Policlinico, Milan, Italy. Patients were included if they had ARDS [19]. Exclusion criteria were age < 18 years, requirement of extracorporeal membrane oxygenation support, presence of barotrauma or hemodynamic instability defined as hypotension with mean arterial pressure < 60 mmHg despite fluid expansion, and vasoactive support. Patient inclusion in this study was slow due, primarily, to the availability of both dedicated staff and the CT lab, and the need to recruit patients early in disease progression where patients could be subjected to recruitment maneuvers.

The study was conducted in accordance with the Declaration of Helsinki and approved by the institutional review board of Ospedale Maggiore Policlinico (approval number #2425). Informed consent to participate in the study was obtained from all patients. In case the patient was not capable of giving informed consent at the time of enrollment, deferred consent was given. As soon as possible thereafter, an investigator provided study information to the patient or their legally designated representative and requested informed consent. In case the patient or legally designated representative did not consent to the study, they were informed of the right to object to the use of study data obtained from the patient.

**Fig. 1:**
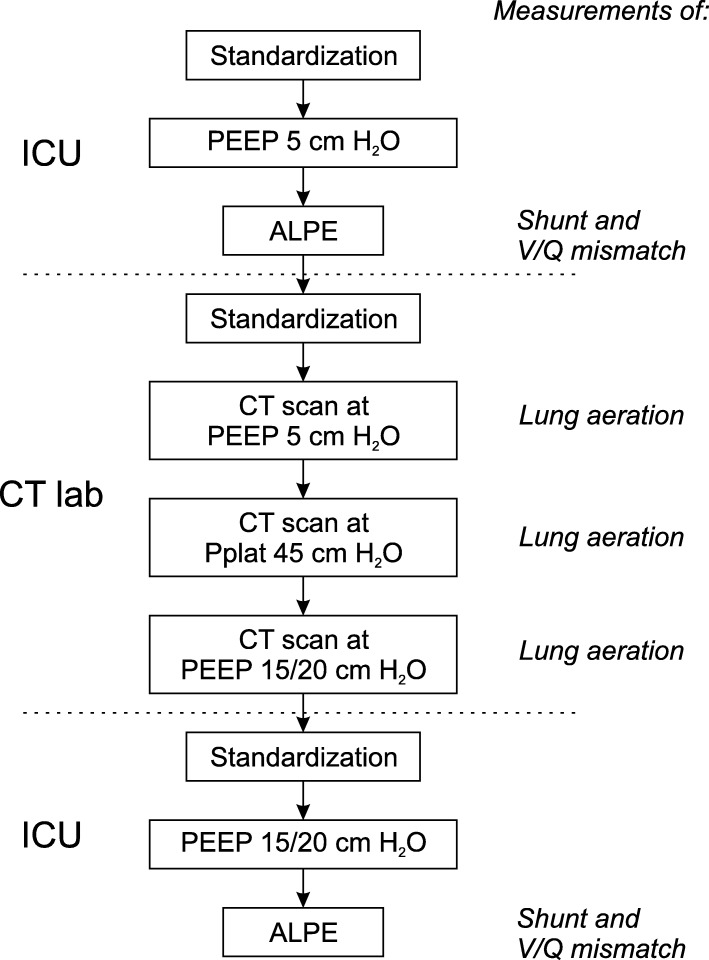
Study protocol. Protocol steps and measurements performed in the protocol. Separation of protocol steps between different locations is indicated (dashed lines). Measurements of shunt and V/Q mismatch were performed in the intensive care unit (ICU). Lung aeration measurements were performed in the CT lab

Figure 1 summarizes the study protocol, indicating which measurements were performed in the intensive care unit and CT lab. Shunt and V/Q mismatch were measured using the automatic lung parameter estimator (ALPE) method [20, 21] (ALPE integrated, Mermaid Care A/S, Nørresundby, Denmark). Lung aeration was assessed by CT scan analysis. Further details on the measurement of shunt, V/Q mismatch, and lung aeration are provided below.

Pulmonary status was standardized three times: (1) initially in the ICU prior to the measurement of shunt and V/Q mismatch at PEEP of 5 cmH_2_O, (2) when arriving at the CT lab prior to performing CT scans, and (3) on returning to the ICU before the measurement of shunt and V/Q mismatch at PEEP of 15 or 20 cmH_2_O. Standardization involved a lung recruitment maneuver comprising 90 s in pressure control ventilation mode with a plateau pressure of 45 cmH_2_O. During recruitment maneuvers, respiratory rate was set to 10 min^−1^, I:E ratio was set to 1:1, and PEEP and FiO_2_ were maintained at levels set at the bedside as part of the routine care. Following standardization, lung-protective ventilation was applied with PEEP set to either low (5 cmH_2_O) or high (15 or 20 cmH_2_O) values as illustrated in Fig. 1. Tidal volume was set to ensure a plateau pressure ≤ 35 cmH_2_O. PEEP levels were maintained 20 min prior to the ALPE measurements. CT scans were performed at three levels: low PEEP of 5 cmH_2_O; high PEEP which was set to 20 cmH_2_O or 15 cmH_2_O if Pplat > 35 cmH_2_O at PEEP of 20 cmH_2_O; and PEEP of 45 cmH_2_O.

At baseline, prior to the initial standardization and before each ALPE, ventilator settings were registered and measurements of arterial blood, arterial blood pressure, and, if available, central venous blood and blood pressure were taken. Cardiac output (CO) was measured at baseline by echocardiography in 9 patients and Swan-Ganz catheter in two patients. CO measurement was not available in one patient.

### Measurement of shunt and V/Q mismatch

The ALPE method measures shunt and V/Q mismatch by estimating the parameters of a physiological model. The method involves modification of inspired oxygen fraction (FiO_2_) in 3–5 steps, the complete procedure taking approximately 10–15 min [20]. At each FiO_2_, the ALPE device automatically detects when a steady state occurs and then measures arterial oxygen saturation (pulse oximetry), end-tidal fractions of oxygen (FETO_2_) and carbon dioxide (FETCO_2_), respiratory rate, tidal volume, oxygen consumption, and carbon dioxide production. A single arterial blood gas sample is required to measure arterial pH, oxygen saturation (SaO_2_), carbon dioxide partial pressure (PaCO_2_), and hemoglobin levels. In this study, arterial blood gas samples were predominantly taken at low levels of oxygenation or near the shoulder of the FETO_2_–SaO_2_ relationship, as measurements at these oxygenation levels provide most information for separating gas exchange problems due to low V/Q mismatch and shunt.

To allow analysis of both oxygen and carbon dioxide gas exchange in parameter estimation, measurement data from the ALPE method were exported and analyzed as previously outlined by Karbing et al. [12]. The analysis consisted of fitting a three-parameter model of oxygen and carbon dioxide exchange to measurement data, by calculating values for model parameters which minimized the difference between model-simulated and measured values of SaO_2_ and PaCO_2_ [12]. In this estimation, anatomical (including apparatus) dead space was assumed to be 150 ml, saturated water vapor pressure was assumed to be 6.3 kPa, and the concentration of 2,3-diphosphoglycerate (CDPG) was estimated from blood gas values using the oxygen dissociation curve as implemented in a model of acid-base chemistry of the blood [22]. CO measurement was available at both low and high PEEP levels in four patients, two by Fick’s principle from Swan-Ganz measurements, and two from echocardiography. In another 9 patients, CO measured by echocardiography at baseline was used for both PEEP levels. CO was assumed to be 6.0 l/min in the patient where a CO measurement was not available, this being the average of the measured baseline CO in the studied patients.

The fitting of the physiological model to measurements yielded three parameters describing pulmonary gas exchange: pulmonary shunt—the part of pulmonary perfusion not reaching ventilated alveoli reported as a percentage of CO; the degree of low V/Q mismatch reported as Δ_A-c_PO_2_—the drop in oxygen partial pressure from alveolar gas to pulmonary capillary blood prior to mixing with shunted venous blood; and degree of high V/Q mismatch reported as Δ_A-c_PCO_2_—the increase in carbon dioxide partial pressure from alveolar gas to lung capillary blood prior to mixing with shunted mixed venous blood. Δ_A-c_PO_2_ is an index of low V/Q mismatch as oxygen exchange is primarily affected by low V/Q. Low V/Q can effectively be negated by oxygen therapy, with the value of Δ_A-c_PO_2_ interpreted as the extra oxygen pressure required in inspired gas to counter low V/Q mismatch. For example, for a barometric pressure of about 100 kPa, a value of Δ_A-c_PO_2_ of 10 kPa can effectively be countered by increasing FiO_2_ by approximately 10%, which raises both alveolar and end-capillary PO_2_ by 10 kPa. Carbon dioxide is primarily affected by high V/Q, and Δ_A-c_PCO_2_ therefore represents an index of high V/Q mismatch. A Δ_A-c_PCO_2_ > 0 kPa can therefore signify a clinical need to increase minute ventilation.

**Fig. 2:**
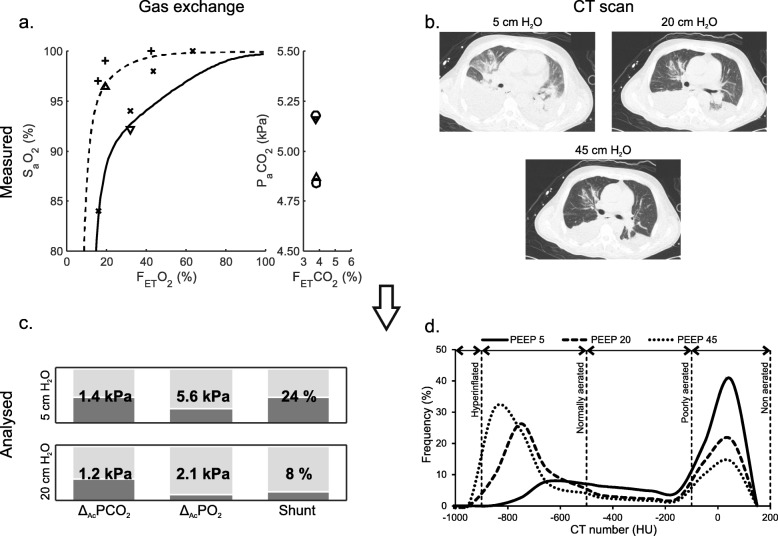
Data analysis example. Patient example of measured data and results of data analysis describing gas exchange and lung aeration. **a** Input data for calculating shunt and V/Q mismatch parameters. Left subplot shows measured FETO_2_ versus SpO_2_ (+) and SaO_2_ at low (▽) and high (△) PEEP along with curves illustrating model-fitted simulations at low (solid curve) and high (dashed curve) PEEP. Right subplot shows measured FETCO_2_ versus simulated PaCO_2_ (○) and measured PaCO_2_ at low (▽) and high (△) PEEP. **b** CT scans taken at 5, 20, and 45 cmH_2_O. **c** Resulting gas exchange model parameters from the model fit to the measured data illustrated in **a**. **d** Resulting CT HU frequency distributions from CT scans illustrated in **b** for PEEP 5 (solid line), 20 (dashed line), and 45 (dotted line) cmH_2_O

Figure 2 illustrates a patient example of measured data and results describing shunt, V/Q mismatch, and lung aeration. Curves showing model-fitted simulations of SaO_2_ and PaCO_2_ are illustrated in Fig. 2a. Figure 2c illustrates the changes in shunt, Δ_A-c_PO_2_, and Δ_A-c_PCO_2_ in a patient example following an increase in PEEP from 5 to 20 cmH_2_O, showing a reduction in shunt and Δ_A-c_PO_2_ but with limited changes in Δ_A-c_PCO_2_. This result is consistent with the observed effect on oxygenation measurements in Fig. 2a, where the FETO_2_–SaO_2_ relationship at low PEEP (solid line) is vertically depressed, indicating an oxygenation problem due to shunt as characterized by the limited response to changes in FiO_2_. The shunt is almost abolished at high PEEP levels (dashed line). In addition, both the difference between FETCO_2_ and PaCO_2_ and the value of Δ_A-c_PCO_2_ show limited changes with PEEP, suggesting no increase in high V/Q mismatch on increasing PEEP.

### CT scan acquisition and analysis

CT scans were performed with Siemens Medical Solution Somaton Definition Flash Syngo CT 2011 (Munich Germany). CT scans were acquired at PEEP 5 and 15/20 cmH_2_O during end-expiratory pause and at 45 cmH_2_O pressure during end-inspiratory pause (not shown). For CT scan analysis, lung profiles were manually delineated, excluding hilar vessels, main bronchi, pleural effusion, as well as areas with edge and partial volume effects. The analysis of segmented images was performed with custom software (Soft-E-Film, http://www.elektron.it, Milan, Italy).

We analyzed lung aeration according to four CT number ranges in Hounsfield units (H) [23]: hyperinflated (− 1000 to − 900 H), normally aerated (− 900 to − 500 H), poorly aerated (− 500 to − 100 H), and non-aerated (− 100 to + 200 H). The total volume of each compartment was reported in the percentage of total lung (voxel) volume. Lung recruitment was calculated using Eq. 1:

1 $$ \mathrm{Recruitment}=\frac{m_{\mathrm{non}-\mathrm{aerated},\kern0.5em \mathrm{PEEP}5}-{m}_{\mathrm{non}-\mathrm{aerated},\kern0.5em \mathrm{PEEP}\mathrm{High}}}{m_{\mathrm{total},\kern0.5em \mathrm{PEEP}5}} $$

where *m*_non-aerated,PEEPHigh_ is the weight of non-aerated tissue at PEEP of 15 or 20 cmH_2_O, and *m*_total,PEEP5_ is the total lung tissue weight at PEEP of 5 cmH_2_O.

Lung tissue weight (*m*_tissue_) was calculated from lung tissue volume (*V*_tissue_) assuming lung parenchyma density is that of water (1000 g/l). *V*_tissue_ was calculated from Eq. 2, assuming total lung volume (*V*_total_) is composed of air and lung parenchyma with average CT numbers of − 1000 H and 0 H, respectively [24]:

2 $$ {V}_{\mathrm{tissue}}=\left[1-\frac{\mathrm{CT}}{-1000\ \mathrm{H}}\right]\times \left({V}_{\mathrm{total}}\right) $$

Figure 2b illustrates CT scans taken at the three PEEP levels for the same patient example as illustrated in the remaining subplots illustrating that in this patient, a PEEP increase from 5 to 20 cmH_2_O reduced the fraction of non-aerated tissue with an increase in normally aerated tissue in line with the improvement in shunt. A small increase in hyperinflated tissue was also observed.

### Statistics

Statistical analysis was performed with SPSS (SPSS Statistics 22.0, IBM). Normality of distributions was assessed with quantile-quantile plots and Shapiro-Wilk test. Normally and non-normally distributed measurements are reported as mean ± SD or median (interquartile range), respectively. Paired *t* test or Wilcoxon’s signed-rank test was performed to compare values at low and high PEEP, as appropriate. Pearson and Spearman rank correlation analysis was performed for correlation between changes in gas exchange (shunt, low and high V/Q mismatch) and changes in lung aeration for normally and non-normally distributed parameters, respectively. Response to PEEP was considered beneficial when increases in PEEP resulted in improvement in gas exchange parameters (lower shunt, Δ_A-c_PO_2_, and Δ_A-c_PCO_2_) and detrimental if PEEP increase caused deterioration in gas exchange parameters (higher shunt, Δ_A-c_PO_2_, and Δ_A-c_PCO_2_).

## Results

Thirteen patients were included in the study, with one patient excluded due to ALPE device technical issues, leaving measurements from 12 patients for data analysis. ALPE and CT measurements were performed in all patients, but the availability of other measurements varied as indicated in the tables. Table 1 lists the baseline values. Patients were diagnosed at admission with pneumonia (n=8), septic shock (n=2), pulmonary contusion (n=1), or spondylodiscitis (n=1).

**Table 1:** Patient baseline characteristics and demographics

Variable	Number	Baseline value
Age (years)	12	56 ± 18
Sex (*n* (%), male)	12	9 (75%)
PaO_2_/FiO_2_ (mmHg)	12	163 ± 60
SaO_2_ (%)	12	94.7 ± 3.0
PaCO_2_ (kPa)	12	5.8 ± 1.1
Respiratory system compliance (ml/cmH_2_O)	11	35 ± 12
FiO_2_ (%)	12	59 ± 16
PEEP (cmH_2_O)	12	11 ± 4
Plateau pressure (cmH_2_O)	11	27 (20–28)
Mean airway pressure (cmH_2_O)	11	16 ± 5
Vt (ml)	12	446 ± 76
Vt (ml/kg IBW)	12	7.4 ± 1.7
Respiratory rate (min^−1^)	12	20 ± 6
Mean arterial pressure (mmHg)	11	78.2 ± 12.2
Central venous pressure (mmHg)	12	13 ± 5
CO (l/min)	11	6.0 ± 1.7
ARDS severity	12	
Mild (*n* (%))		3 (25%)
Moderate (*n* (%))		8 (67%)
Severe (*n* (%))		1 (8%)
BMI (kg/m^2^)	12	25.8 ± 7.2
SAPS II at admission	12	37 ± 9
SOFA 24 h from admission	12	8 ± 2
Duration of mech. vent. at study (days)	12	1.0 (1.0–2.0)
Total duration of mech. vent. (days)	12	6.0 (5.0–18.5)
ICU length of stay (days)	12	14.0 ± 9.3
ICU mortality (*n* (%))	12	4 (33%)

Table 2 reports ventilator settings, respiratory mechanics, hemodynamics, and gas exchange measured after standardization at PEEP 5 cmH_2_O and following an increase in PEEP of 15 or 20 cmH_2_O. Half of the patients tolerated PEEP of 20 cmH_2_O with remaining patients subjected to 15 cmH_2_O. Plateau pressure (Pplat), mean airway pressure, mean arterial pressure, and PaO_2_/FiO_2_ ratio increased significantly with PEEP, with shunt decreasing significantly (Table 2).

**Table 2:** Ventilator settings, respiratory mechanics, hemodynamics, and gas exchange at low and high PEEP

Physiological variable/parameter	Number	PEEP, 5 cmH_2_O	PEEP, 15/20 cmH_2_O	*p* value*
FiO_2_ (%)	12	50 (43–68)	50 (40–50)	0.066
No. of patients at PEEP 20 cmH_2_O	12	–	6 (50%)	NA
Respiratory rate (min^−1^)	12	23 ± 7	23 ± 7	0.593
Vt (ml)	12	415 ± 98	409 ± 85	0.593
Vt (ml/kg IBW)	12	6.9 ± 1.9	6.8 ± 1.7	0.593
Pplat (cmH_2_O)	11	17 (16–19)	31 (28–35)	0.003
Driving pressure (cmH_2_O)	11	12 (11–14)	12 (11–16)	0.754
Mean airway pressure (cmH_2_O)	11	9 ± 1	22 ± 3	< 0.001
Resp. system compliance (ml/cmH_2_O)	11	34 ± 10	34 ± 13	0.989
Lung compliance (ml/cmH_2_O)	7	39 ± 14	40 ± 24	0.970
Chest wall compliance (ml/cmH_2_O)	7	180 (120–275)	310 (140–430)	0.138
Heart rate (1/min)	11	86 ± 20	92 ± 16	0.111
Mean arterial pressure (mmHg)	11	74.8 ± 12.2	80.6 ± 11.6	0.014
Central venous pressure (mmHg)	11	11.6 ± 4.8	13.2 ± 4.9	0.062
ScvO_2_ (%)	10	71.3 ± 6.2	73.3 ± 7.9	0.362
VO_2_ (ml/min)	12	229 ± 68	268 ± 78	0.060
VCO_2_ (ml/min)	12	211 ± 54	232 ± 47	0.209
PaO_2_/FiO_2_ (mmHg)	11	130 ± 58	220 ± 82	0.003
SaO_2_ (%)	11	89.8 ± 5.2	94.0 ± 4.8	0.098
PaCO_2_ (kPa)	11	5.5 (5.2–6.7)	5.8 (5.3–9.0)	0.155
CDPG (mmol/l)	12	5.2 ± 0.8	5.3 ± 0.8	0.866
Δ_A-c_PCO_2_ (mmHg)	12	7.9 (6.9–13.8)	7.3 (3.9–15.7)	0.735
Δ_A-c_PO_2_ (mmHg)	12	34(14–132)	37 (17–58)	0.156
Δ_A-c_PCO_2_ (kPa)	12	1.05 (0.93–1.85)	0.98 (0.52–2.09)	0.735
Δ_A-c_PO_2_ (kPa)	12	4.6 (1.8–17.6)	5.0 (2.2–7.8)	0.156
Pulmonary shunt (%)	12	33 ± 15	22 ± 14	0.020

*ScvO*_*2*_ central venous oxygen saturation

*p value from paired *t* test or Wilcoxon signed-rank test

Table 3 summarizes the changes in CT scan variables between low and high PEEP. Non-aerated lung tissue weight and fraction decreased with increasing PEEP, while normally aerated and hyperinflated tissue fractions increased with PEEP, with only marginal changes observed in hyperinflated tissue.

**Table 3:** CT scan variables at 5 and 15/20 cmH_2_O

CT scan variable	Number	PEEP, 5 cmH_2_O	PEEP, 15/20 cmH_2_ O	*p* value*
Total lung weight (g)	12	1887 ± 396	1884 ± 315	0.951
Non-aerated lung weight (g)	12	1152 ± 458	790 ± 425	0.008
Recruitment (%)	12	–	18 ± 17	–
Hyperinflated tissue (%)	12	0 (0–0)	0 (0–0)	0.006
Normally aerated tissue (%)	12	25 ± 12	45 ± 12	< 0.001
Poorly aerated tissue (%)	12	26 (22–35)	29 (19–33)	0.538
Non-aerated tissue (%)	12	45 ± 15	25 ± 14	0.002

*p value from paired *t* test or Wilcoxon signed-rank test

**Fig. 3:**
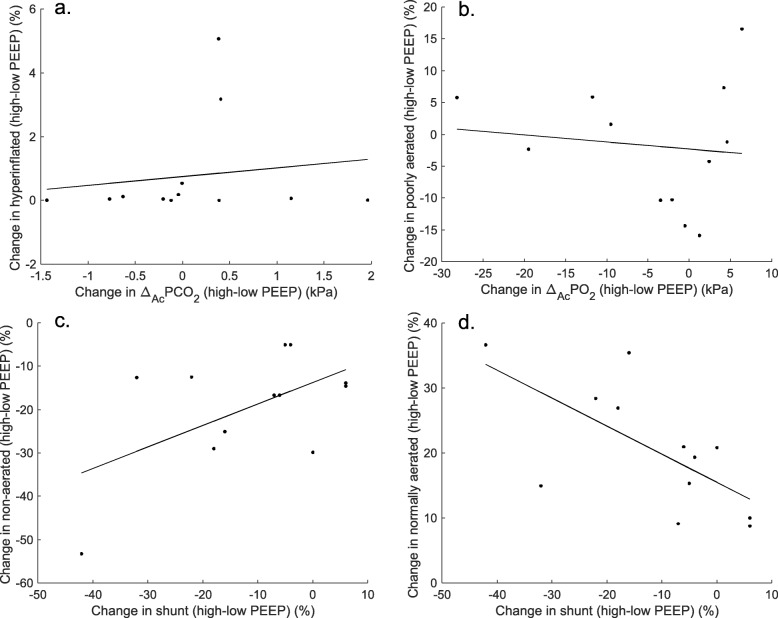
Correlation of lung aeration and shunt and V/Q mismatch. Scatterplots of changes in gas exchange parameters and CT scan lung aeration with increases in PEEP (values at high minus low PEEP) and linear regression lines (solid lines). **a** Changes in Δ_A-c_PCO_2_ versus changes in hyperinflated lung regions. **b** Changes in Δ_A-c_PO_2_ versus changes in poorly aerated lung regions. **c** Changes in shunt versus changes in non-aerated lung regions. **d** Changes in shunt versus changes in normally aerated lung regions

Changes from low to high PEEP in Δ_A-c_PCO_2_ were not significantly correlated with changes in the percentage of hyperinflated lung regions (*ρ* = 0.224, *p* = 0.484, Fig. 3a) or normally aerated lung regions (*r* = − 0.316, *p* = 0.317, not shown). Changes in Δ_A-c_PO_2_ were not significantly correlated with changes in poorly aerated lung regions (*r* = − 0.120, *p* = 0.710, Fig. 3b) or normally aerated lung regions (*r* = 0.315, *p* = 0.318, not shown). Changes in shunt were not correlated with changes in non-aerated lung regions (ρ = 0.235, *p* = 0.463, Fig. 3c) but significantly correlated with changes in normally aerated lung regions (*r* = − 0.665, *p* = 0.018, Fig. 3d). Shunt and low and high V/Q mismatch changes from PEEP 5 to 20/15 cmH_2_O were not correlated with recruitment at 20/15 cmH_2_O (*p* > 0.05, not shown).

**Fig. 4:**
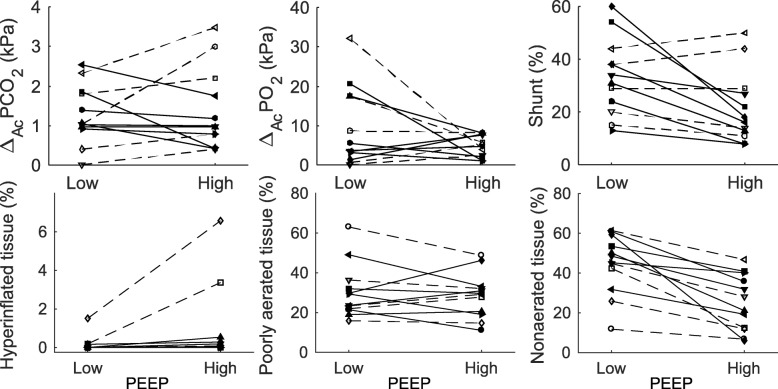
Individual patient changes in shunt, V/Q mismatch, and lung aeration. Changes from low to high PEEP in shunt and V/Q mismatch (top row) and lung aeration (bottom row). Beneficial and detrimental responses in shunt and V/Q mismatch to an increase in PEEP are marked by line styles, with solid and dashed lines signifying beneficial and detrimental responses, respectively. The combinations of symbols and line styles are unique per patient so that individual patients can be identified across all subplots

Seven patients showed a beneficial response to an increase in PEEP with a decrease in pulmonary shunt and some or limited decrease in Δ_A-c_PCO_2_ (Fig. 4 top row, solid lines). These patients showed consistent decreases in the percentage of non-aerated lung regions and no change in percentage of hyperinflated lung regions, but with no clear pattern in poorly aerated regions (Fig. 4, bottom row). Overall, these patients responded beneficially to increases in PEEP both according to shunt, V/Q mismatch, and aeration. Figure 2 illustrates shunt, V/Q mismatch, and aeration measurements for a typical patient showing a beneficial response to an increase in PEEP.

The remaining 5 patients showed two different patterns of detrimental response to increase in PEEP (dashed lines Fig. 4). One patient showed a decrease in shunt but a marked Δ_A-c_PCO_2_ increase (open circles and dashed line). Four patients showed increases in both Δ_A-c_PCO_2_ and shunt (remaining open symbols). The percentage of non-aerated lung regions decreased in all these patients on increasing PEEP, but with hyperinflation observed in 2 of the patients with a detrimental response.

## Discussion

This study investigated whether increasing PEEP in patients with ARDS resulted in changes in shunt and V/Q mismatch which were related to changes in lung aeration. The observed effects of the increase in PEEP on shunt [3, 4, 9, 11] and lung aeration [3, 25, 26] were similar to previously reported results. The observed changes in lung aeration were similar to those reported by Gattinoni and co-authors for the subgroup of patients in their study with a higher percentage of recruitable lung [1]. This subgroup appears similar to the patients in this study with recruitment of up to 35% when changing PEEP from 5 to 15 cmH_2_O and with little to no hyperinflation at high PEEP. The subgroup described by Gattinoni et al. also had similar PaO_2_/FiO_2_ ratios at baseline, admission diagnoses primarily due to pneumonia, and average total lung weights around 1800 g.

To the best of the authors’ knowledge, this is the first analysis comparing the bedside, model-based estimation of shunt and V/Q parameters with lung aeration following PEEP changes during lung-protective ventilation in ARDS patients.

Only changes in pulmonary shunt and normally aerated tissue were significantly correlated. However, shunt was not always reduced with an increase in PEEP, despite all patients showing an increased percentage of normally aerated tissue and reduced percentage of non-aerated tissue. The correlation between changes in shunt and normally aerated tissue is therefore perhaps surprising; however, similar findings have been reported previously. Gattinoni et al. reported a correlation between shunt and normally aerated and non-aerated tissue changes [3]. Brunet et al. only demonstrated a significant correlation between non-aerated tissue and oxygenation changes when excluding a patient showing oxygenation deterioration [25]. In contrast, several studies have found a weak but significant correlation between shunt and non-aerated lung changes [3, 27, 28] or %atelectasis [29] and weak to moderate correlation between oxygenation and non-aerated lung [25, 28, 30–32]. A single study by Borges et al. reported, however, a good correlation between oxygenation and %collapse [33].

Overall, the presented results on changes in shunt and V/Q mismatch with PEEP during lung-protective ventilation suggest that the response in gas exchange to increase in PEEP is heterogeneous, as previously indicated in studies applying MIGET to measure the response to PEEP changes in ARDS in the “high Vt-era” [9, 11]. The results also indicate that changes in lung volume (aeration) do not always go hand in hand with changes in ventilation and perfusion. We therefore investigated individual patient response to PEEP in values of shunt and V/Q mismatch and found three distinct patterns of response. The association between changes in lung aeration and changes in shunt and V/Q mismatch will therefore be discussed in the context of these three patterns of response.

Increasing PEEP may be beneficial as indicated by a decrease in pulmonary shunt without worsening high V/Q mismatch, as observed in seven patients. This was consistent with observations of improved aeration without hyperinflation. Two non-exclusive mechanisms may reduce shunt, these being the recruitment of lung units increasing end-expiratory lung volume or decrease in CO leading to less perfusion of unventilated lung areas, the latter also termed vascular derecruitment [9, 10]. The observation of an increase in normally aerated and decrease in non-aerated lung regions following PEEP increase in these seven patients was consistent with lung recruitment being responsible for shunt improvement.

Increasing PEEP may be detrimental as indicated by an increase in high V/Q mismatch and either reduction or increase in shunt as seen in one and four patients, respectively. Only two patients showing an increase in high V/Q mismatch also showed an increase in hyperinflation according to the CT aeration analysis. MIGET studies have previously shown an increase in both shunt and high V/Q mismatch following an increase in PEEP [9, 11]. An increase in venous admixture has similarly been shown following an increase in PEEP [3]. As increasing PEEP caused a decrease in non-aerated tissue in all patients, the observed increase in shunt in four patients can most likely be explained by no perfusion of otherwise adequately aerated regions of the lung. This could be caused by the redistribution of blood flow toward the dependent atelectatic regions of the lung due to elevated intrathoracic pressure following the PEEP increase [34]. The discrepancy between detection of hyperinflation from aeration and measurement of high V/Q mismatch by Δ_A-c_PCO_2_ may be due to the different effect of PEEP increase on end-expiratory aeration as measured by CT in the present study and changes in distributions of ventilation and perfusion, which in concert determine the effect of PEEP on V/Q mismatch. Perfusion redistribution toward dependent lung regions could contribute to such discrepancy by reducing perfusion to non-dependent high V/Q regions without these necessarily being hyperinflated. Ventilation may also be redistributed toward dependent lung regions, as observed on increasing PEEP in studies with electrical impedance tomography [35, 36], and EIT-based estimations of alveolar hyperdistension have been proposed [37]. As such, the hyperinflation detected by CT aeration may be in regions with reduced ventilation. However, the observed increase in high V/Q mismatch in some of the patients of the present study indicates that the overall redistributions of perfusion and ventilation following the PEEP increase have been poorly matched in these patients.

We observed no distinct pattern of Δ_A-c_PO_2_ changes with an increase in PEEP, in line with MIGET studies [9, 11] indicating large variation in the effect of PEEP increase on low V/Q mismatch. Low V/Q mismatch may be increased despite lung recruitment as recruited lung units may become low V/Q units. This can be due to the separate or combined effects of regional hyperperfusion and reduced ventilation caused by airway constriction or secretion [38].

The purpose of this study was to compare changes in shunt and V/Q mismatch with changes in lung aeration following an increase in PEEP. We have performed this comparison between two PEEP levels of 5 cmH_2_O and either 15 or 20 cmH_2_O to evaluate if the two measurement modalities would give similar quantification of recruitment. Our results should therefore not be construed as indications for a proposed strategy for optimizing PEEP but, given the three observed patterns of gas exchange response to PEEP, rather as a support for individual PEEP titration. Different strategies have been proposed for individual PEEP titration [5, 33]. In their maximal recruitment strategy, Borges et al. performed recruitment maneuvers of incremental Pplat between 40 and 60 cmH_2_O followed by a decremental PEEP titration targeting a combined index of PaO_2_+PaCO_2_ [33]. This strategy, primarily targeting oxygenation, led to a good correlation between repeated measurements of lung collapse assessed by CT aeration and PaO_2_ across recruitment maneuvers and titrated PEEP. Strategies for determining optimal PEEP have also been suggested to focus on lung mechanics. Chiumello et al. compared 3 mechanics-oriented strategies with an oxygenation-oriented “open-lung” strategy [5]. They found that PEEP selected according to lung mechanics were unrelated to lung recruitability assessed by CT-measured lung aeration, whereas the oxygenation-oriented strategy led to PEEP levels related to recruitability. Whether individually titrated PEEP values can achieve both improvements in aeration, shunt and V/Q mismatch remains to be determined. The results of Chiumello et al.’s and Borges et al.’s study indicate that PEEP strategies targeting oxygenation are preferable for improving aeration, and the results presented by Borges et al. are particularly encouraging showing that individually titrated PEEP can improve oxygenation, lung mechanics, and lung collapse, indicating that a good compromise between redistributing ventilation and perfusion and protecting the lungs can be achieved. Their reported increase in oxygenation indicates improvement in shunt, and their use of FiO_2_ of 100% would effectively counter low V/Q mismatch. It would be interesting to investigate whether this strategy would result in a beneficial response for a high V/Q mismatch.

Our study has some limitations. We did not measure CO at both low and high PEEP in all patients and cannot rule out that some changes in shunt were due to changes in CO. However, it has previously been demonstrated that ALPE shunt measurements are insensitive to CO variation of ±2 l/min [39]. While a statistically significant increase in mean arterial pressure was observed from PEEP 5 to 15/20 cmH_2_O, the magnitude of this change was less than the previously observed with similar PEEP changes in ARDS patients by Borges et al. who reported a decrease in the average cardiac index of less than 1.0 l min^−1^ m^2^ [33]. According to Dantzker et al. [40], a decrease in CO can cause a reduction in shunt. If CO decrease played an important part in reducing shunt in this study, then oxygen delivery and tissue oxygenation would be expected to decrease with an increase in PEEP at constant or reduced FiO_2_ and constant minute ventilation. Central venous oxygen saturation showed a trend to increase with PEEP, and although not significant, this in combination with the trend of increasing oxygen consumption indicates that a decrease in CO was not the predominant mechanism for the observed shunt reduction from low to high levels of PEEP.

The model-based estimation of shunt and V/Q mismatch applied in the present study includes some assumptions and simplifications, which are important to discuss. These are the use of a guessed value of anatomical dead space; omission of diffusion limitation effects; estimation of parameters from a procedure where FiO_2_ is titrated; and the use of two V/Q compartments and a shunt compartment to represent pulmonary gas exchange. We will now discuss these four issues in turn.

Anatomical dead space including apparatus dead space was assumed to be 150 ml across PEEP levels and subjects. Anatomical dead space is reduced in endotracheal intubated patients to an expected range of 33–99 ml [41]. However, the apparatus dead space can add an additional 60 ml due to filter and 55 ml due to respiratory tubes [42], reflected in our general guess of 150 ml. PEEP modifications have previously resulted in only marginal changes in anatomical dead space [17]. Anatomical dead space should not be confused with physiological dead space, which includes alveolar dead space [43] and has been shown to vary significantly with PEEP [9–11]. Alveolar dead space is composed of alveoli receiving ventilation but no perfusion, resulting in the extremely high V/Q of infinity. This part of the physiological dead space will vary with changes in ventilation and perfusion due to PEEP modifications. The Δ_A-c_PCO_2_ parameter estimated to describe a high V/Q mismatch in this study encompasses the effects of high V/Q mismatch including that of alveolar dead space.

The effect of diffusion limitation on gas exchange is similar to that of V/Q mismatch, that is, a difference in partial pressures of O_2_ and CO_2_ between alveolar air and lung capillary blood [44]. However, studies with MIGET have shown that V/Q mismatch is likely a better representation of the physiology in the majority of cases, such that including diffusion limitation is rarely a requirement to accurately describe pulmonary gas exchange [10, 45], with the exceptions of pulmonary fibrosis [45], exercise [46], or mild exercise during hypoxia [46, 47]. In ARDS patients and patients with severe pneumonia, MIGET predictions disregarding diffusion limitation have shown close agreement with measured PaO_2_ indicating that diffusion limitation is not important to describe pulmonary gas exchange [10].

Parameters describing pulmonary shunt and V/Q mismatch have been estimated in this study by fitting a physiological model to measurements of end-tidal expired gasses, metabolism, and blood gas values at varied levels of FiO_2_, where the FiO_2_ titration allows separation of the effects of shunt and V/Q mismatch [48]. Experimentally, this process requires a fast oxygen analyzer and capnograph for measuring end-tidal expired levels of O_2_ and CO_2_. Such devices are not always used in clinical practice but are readily available. A further assumption of this method is that FiO_2_ variation does not affect lung physiology. Changes in FiO_2_ may affect lung physiology through reabsorption atelectasis [49] and hypoxic pulmonary vasoconstriction (HPV). Reabsorption atelectasis should not affect parameter estimation as shunt, and V/Q mismatch parameters can be estimated from FiO_2_ levels lower than 0.8 [13], which have been shown to cause minimal atelectasis during induction of anesthesia [50]. HPV inhibition can affect pulmonary gas exchange at a moderate [51] or considerable extent [52]. However, HPV inhibition in these studies constitutes maximal responses to changes in FiO_2_. The smaller FiO_2_ steps performed in this study to estimate shunt and V/Q mismatch model parameters are less likely to have an effect on HPV and pulmonary gas exchange. Studies with the MIGET technique [53, 54], computer simulations [55, 56], and a study with both the MIGET technique and an oxygen gas exchange model [18] have shown limited changes in shunt and V/Q parameters with large variations in FiO_2_. The effects of HPV on parameter estimation are therefore likely to be limited [17].

The applied physiological model gives an integrated view of pulmonary gas exchange based on two ventilated and perfused gas exchanging compartments and one shunt compartment. The MIGET would provide greater resolution with a model incorporating 50 compartments of varying V/Q [8] but is considered too costly for routine clinical use [57]. The model used for estimating gas exchange parameters in the present study has previously been shown to agree well with MIGET gas exchange measurements of retention, excretion, and oxygenation in animal acute lung injury models [17, 18]. The concordance of our results with MIGET studies of PEEP in ARDS [9, 11], suggests a possible bedside role of the ALPE technique in evaluating PEEP effects. The feasibility of doing so has been demonstrated by its application in prospective studies for understanding the effect of mechanical ventilation including PEEP on pulmonary gas exchange during surgery [15, 16].

Our study was of a preliminary nature, and future larger studies are necessary to further explore patterns of response in shunt and V/Q mismatch to PEEP modification, as well as the association between these responses, those describing lung aeration and patient outcome. Such studies are required when applying different strategies for selecting PEEP.

## Conclusions

The results show that an improved aeration of the lungs following an increase in PEEP is not always consistent with reduced shunt and V/Q mismatch. We observed three distinct responses in shunt and V/Q mismatch to increases in PEEP: reduction in pulmonary shunt without increasing high V/Q mismatch, increase in both shunt and high V/Q mismatch, and reduction in shunt but increasing high V/Q mismatch, the latter response making PEEP adjustment a balance between improving O_2_ exchange and preserving CO_2_ exchange. Poorly matched redistributions of ventilation and perfusion, between dependent and non-dependent regions of the lung, may explain why some of the patients showed detrimental changes in shunt and V/Q mismatch in response to an increase in PEEP, despite improved aeration.

## Data Availability

The datasets used and/or analyzed during the current study are available from the corresponding author on reasonable request.

## Abbreviations

ALPE: Automatic lung parameter estimator
ARDS: Acute respiratory distress syndrome
CO: Cardiac output
CT: Computed tomography
Δ_A-c_PO_2_: The drop in oxygen pressure from alveolar gas to pulmonary capillary blood prior to mixing with shunted venous blood
Δ_A-c_PCO_2_: The increase in carbon dioxide partial pressure from alveolar gas to lung capillary blood prior to mixing with shunted mixed venous blood
FETO_2_: Gas fraction of oxygen in end-tidal expired gas
FETCO_2_: Gas fraction of carbon dioxide in end-tidal expired gas
FiO_2_: Gas fraction of oxygen in inspired gas
H: Hounsfield unit
MIGET: Multiple inert gas elimination technique
PaCO_2_: Partial pressure of carbon dioxide in arterial blood
PaO_2_/FiO_2_: The ratio of partial pressure of oxygen in arterial blood to inspired oxygen fraction
Pplat: Plateau pressure
PEEP: Positive end-expiratory pressure
SaO_2_: Oxygen saturation of arterial blood
ScvO_2_: Oxygen saturation of central venous blood
V/Q: Ventilation/perfusion ratio
Vt: Tidal volume
